# Polymorphisms in BER genes and risk of breast cancer: evidences from 69 studies with 33760 cases and 33252 controls

**DOI:** 10.18632/oncotarget.23804

**Published:** 2018-01-02

**Authors:** Lele Qiao, Xiaoshan Feng, Gongping Wang, Bo Zhou, Yantong Yang, Mengxiang Li

**Affiliations:** ^1^ The First Affiliated Hospital and College of Clinical Medicine of Henan University of Science and Technology, Luoyang, 471003, China; ^2^ Henan University of Science and Technology, LuoYang, Henan, 471023, China

**Keywords:** BER gene, breast cancer, polymorphism, meta-analysis

## Abstract

Recently, numerous studies have reported an association between single nucleotide polymorphisms in base-excision repair genes and the risk of developing breast cancer, however there is no consensus. The aim of this meta-analysis was to review and quantitatively assess the relationship between single nucleotide polymorphisms in base-excision repair genes and breast cancer risk. The results suggested that a mutation of T to G in rs1760944 may lead to a higher risk of developing breast cancer in the Mongoloid population, and G to A of rs25487 significantly reduced the risk of breast cancer in Mongoloid and Caucasoid populations. In contrast to the CC and CG genotypes, the GG genotype of rs1052133 located on theOGG1 gene appeared to be a protective factor against developing breast cancer in both Mongoloid and Caucasoid populations. There was no evidence to suggest that rs25489, rs1799782, rs1130409, rs1805414 and rs1136410 were associated with breast cancer risk. In conclusion, this study provides evidence to support the theory that DNA repair genes are associated with breast cancer risk, providing information to further understand breast cancer etiology. and The potential biological pathways linking DNA repair, ethnic background, environment and breast cancer require further investigation.

## INTRODUCTION

-Breast cancer (BC) affects about 12% of women worldwide. Statistics indicate that almost 3 million women suffers from BC in 2015 in the United States [1]. Risk factors for developing breast cancer include unhealthy lifestyles, other medical conditions and genetic susceptibility [2, 3]. Epidemiologic studies suggest that women with family history of BC could be more vulnerable to develop BC cancer than those not [4, 5].

Environmental factors and metabolic processes are the two main causes of DNA damage. Ionizing radiation has been confirmed as an environmental risk factor for the development of cancer, which can cause DNA damage of different kinds.[6] Base-excision repair (BER), one of DNA repair pathways, mainly repairs single base in damaged DNA molecule. Mutations occurred in BER related genes can lead to change its repair function, and then increases the probability of developing cancer greatly [7, 8].

Numerous studies have widely explored the relationship between susceptibility to BC and single nucleotide polymorphisms (SNPs) in Base-excision repair genes. However, the conclusions remain indecisive as a result of insufficient samples and/or race diversity. These studies include human apurinic/apyrimidinic endonuclease (APE1), x-ray repair cross-complementing 1 (XRCC1), human 8-oxoguanine DNA glycosylase (OGG1, also known as hOGG1), and poly (ADP-ribose) polymerase-1 (ADPAT1, also known as PARP1). Among these, the relationships between risk of BC and mutations of rs25487 and rs1799782 on the XRCC1 gene have caused the greatest controversy among researchers. Positive association between rs25487 mutation [9–14] and the risk of BC have been reported in single race population studies, but others did not even in the same race [15–21]. Most studies suggest that the rs1799782 mutation is not associated with developing BC, while other studies report it is positively correlated. [9, 11–15, 17, 19] Inconsistent results have also been reported in studies using populations consisting of mixed race. Duell *et al.* (2001) reported a positive association for rs25487 with BC was found among African Americans but not Caucasian Americans [22]. VIEIRAL *et al.* (2015) demonstrates genetic background can influence BC developing, even an inverse association [23].

Meta-analysis is an authoritative way to improve authenticity and provides quantitative pooled values for different races. Previous meta-analysis studies adopted the continental location as the classification standard to discuss the relationship between susceptibility to BC and SNPs in BER genes [24, 25]. However, there are numerous races distributed on the same continent. For example, the Mongoloid race is mainly located in the East and Southeast of Asia, and the Caucasoid race mainly located in Europe, the Americas, Oceania, North Africa, South and West Asia. Therefore, using human race as the standard of classification to use in stratification analysis may be more appropriate. The purpose of this study is to discuss the relationship between risk of developing BC and BER genes based on genetic ancestry.

## MATERIALS AND METHODS

### Literature search strategy

**Figure 1:**
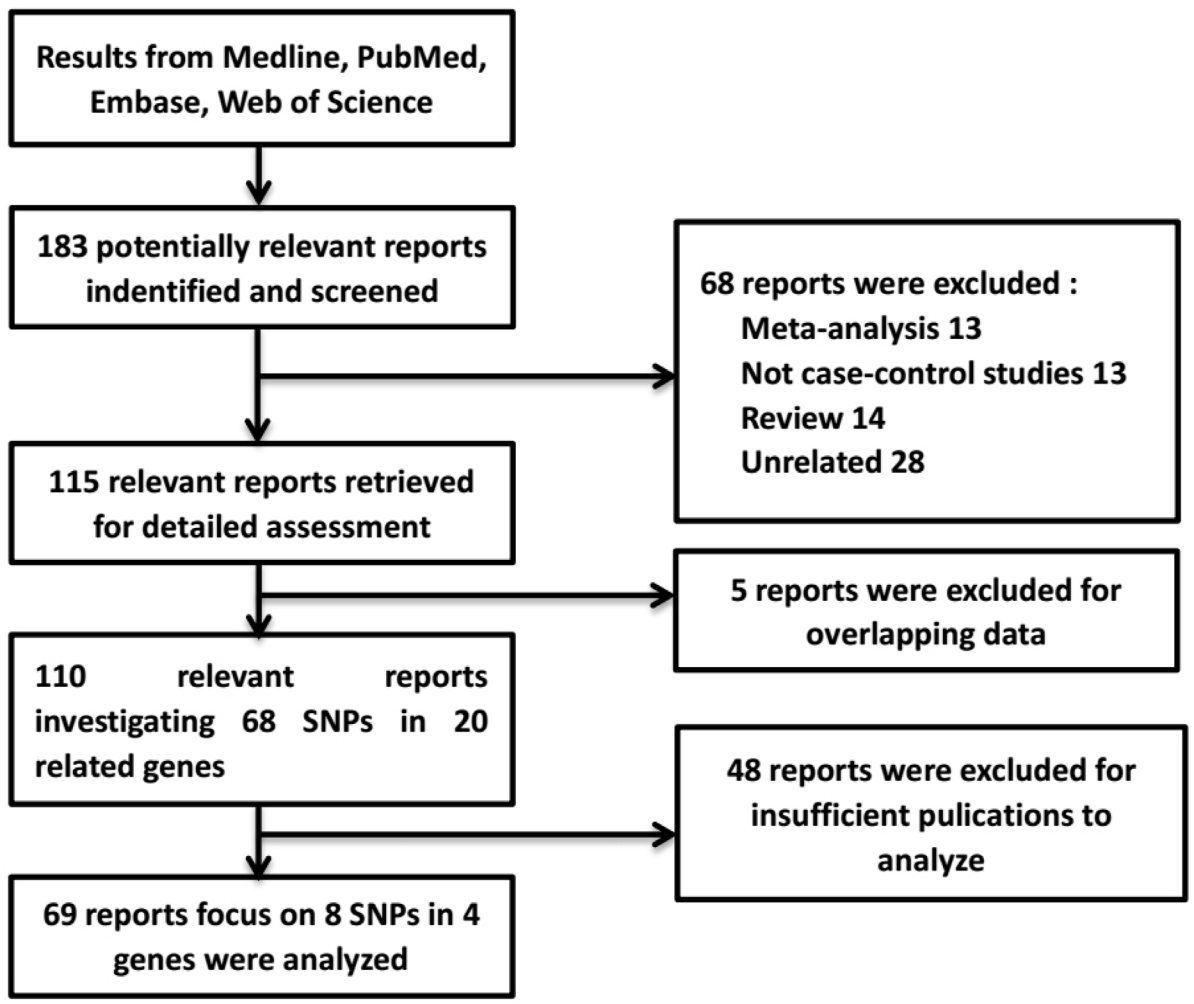
A flow diagram of the studies selection process

The Medline, PubMed, Embase, Web of Science were searched (the last search was updated on October 20th 2016) using the search terms ‘‘breast cancer’’, ‘‘polymorphism’’ or “SNP”, ‘‘DNA Repair Gene’’ or “base excision DNA repair gene” or “BER gene”. All searches were retrieved and their references were checked for other relevant publications. Only published studies with full-text articles were included. When more than one of the same patient populations was included in several studies, only the study with the largest sample size or the complete study was used for this meta-analysis. A flow diagram of the study selection process was shown in Figure 1.

### Inclusion and exclusion criteria

The inclusion and exclusion criteria were established on the basis of discussion and consensus. The inclusion criteria for studies were as follows: (1) case-control studies; (2) the aim was to examine the association of the polymorphisms in BER genes with susceptibility of breast cancer; (3) data provided met the requirements of meta-analysis method; (4) genotype distribution in healthy controls complied with the Hardy–Weinberg equilibrium (HWE).

The exclusion criteria were as follows: (1) did not fit the diagnostic criteria; (2) animal study; (3) the aim of the study didn`t focus on susceptibility to breast cancer or not BER genes; (5) genotype distribution healthy controls deviated from the HWE.

### Racial classification

According to the Meyers Konversations-Lexikon (1885–90), human beings can be divided into three major races: Mongoloid race, Caucasoid race, and Negroid race. The Mongoloid race is a term used for all or some people who are indigenous to East Asia, Central Asia, Southeast Asia, North Asia, South Asia, the Arctic, the Americas, the Pacific Islands and other lesser regions, and are the minority group worldwide (https://en.wikipedia.org/wiki/Mongoloid). The Caucasoid race usually includes some or all of the ancient and modern populations of Europe, the Caucasus, Asia Minor, North Africa, the Horn of Africa, Western Asia, Central Asia and South Asia (https://en.wikipedia.org/wiki/Caucasian_race#cite_note-Pickering-8). The Negroid race populations are found in most of Sub-Saharan Africa and isolated parts of Southeast Asia (Negritos) (https://en.wikipedia.org/wiki/Negroid).

The information included in this meta-analysis was arranged and divided into Mongoloid population, Caucasoid population and Negroid population using the criteria above. If the racial origin of the samples could not be clearly defined, the data was assigned to a mixed race group.

### Statistical analysis

A χ^2^ test was used to determine if observed frequencies of genotypes corresponded to the HWE. Statistical analysis was conducted using R software and a *P*-value ≤ 0.05 was considered statistically significant. Dichotomous data was presented as the odds ratio (OR) with a 95% confidence interval (CI). Statistical heterogeneity was measured using the Q-statistic (P ≤ 0.10 was considered to be representative of statistically significant heterogeneity). Effect of heterogeneity quantified by the *I*^2^ statistic, a fixed effects model was used when there was no heterogeneity in the results of the trials; otherwise, the random effects model was used. Egger’s weighted regression method were used to statistically assess the publication bias (*P* ≤ 0.05 was considered to indicate statistically significant publication bias). The methods of “Influence analysis” and “Trim and Filled analysis” were both conducted to investigate the sensitivity of the pooled ORs.

## RESULTS

183 relevant studies with 69 SNPs on 20 BER related genes were retrieved in this research. 114 articles describing 19 genes were excluded from this study due to insufficient publication number (not more than 3 researches). Finally, this study included 69 papers with 33760 BC cases and 33252 controls, and the information was summarized in Table 1. The flow process was shown in Figure 1.

**Table 1:** Summary of the SNPs studied in this meta-analysis

Genes in BER pathway	SNPs	n. of studies included	n. of Cases	n. of Controls	References included
XRCC1	rs1799782	33	14991	15624	[9, 10, 12, 13, 16–23, 26–46]
	rs25487	47	20995	22964	[9–13, 15–22, 26, 27, 29–45, 47–60]
	rs25489	10	7509	7403	[16, 19, 21, 28, 32, 34, 36, 43, 47, 57]
ADPRT1	rs1805414	3	236	269	[61–63]
	rs1136410	7	3128	2805	[28, 32, 53, 61, 62, 64, 65]
APEX1	rs1130409	12	5154	5858	[13, 14, 28, 32, 35, 36, 51, 66–70]
	rs1760944	4	1415	1827	[13, 14, 68, 69]
OGG1	rs1052133	16	11038	12799	[14, 32, 34, 36, 44, 46, 57, 67, 71–78]

**Figure 2:**
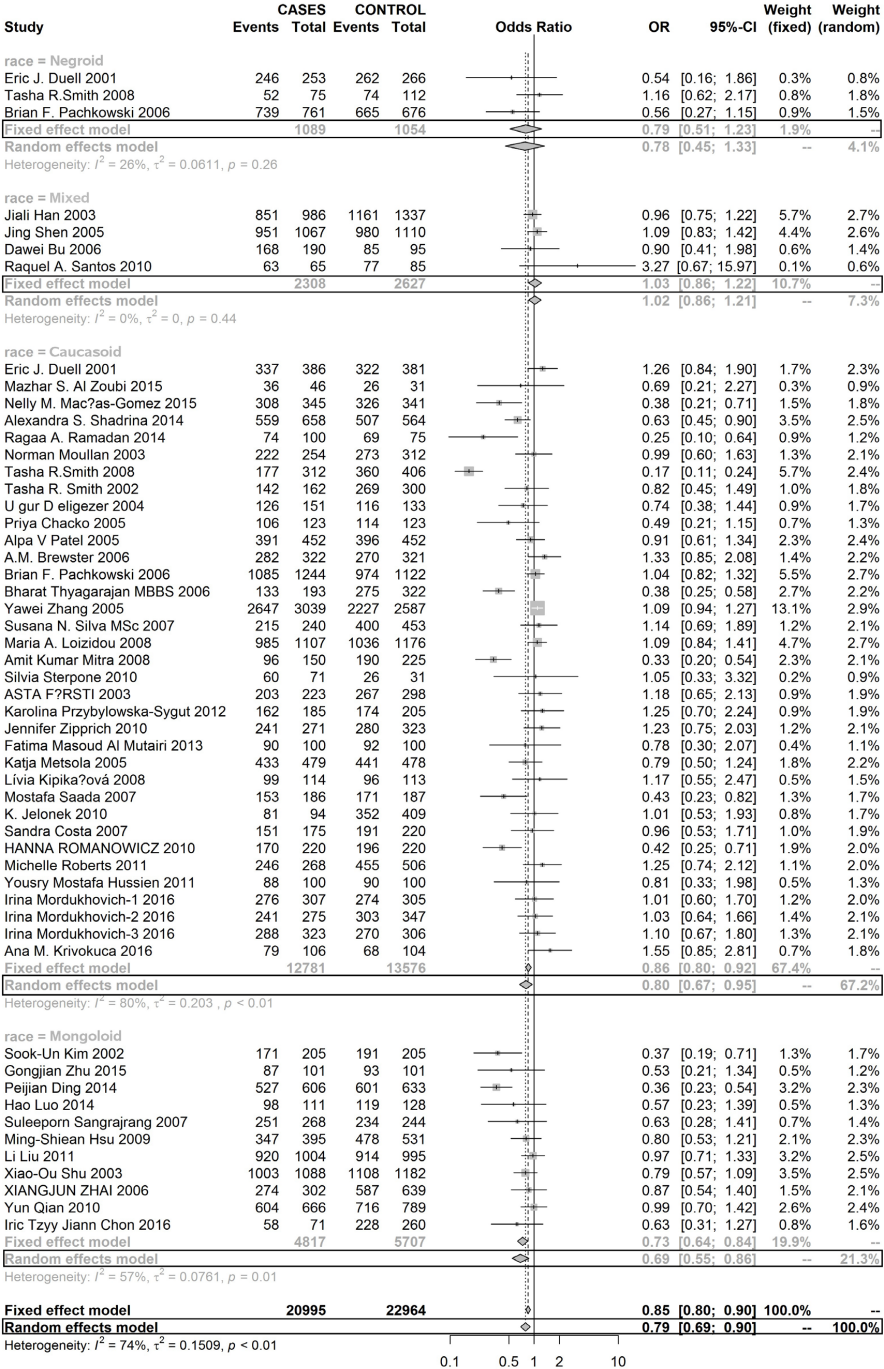
Forest plot of RS25487 polymorphism in XRCC1 and risk to breast cancer (GA+GG vs. AA) (the model adopted was marked by black frame)

**Figure 3:**
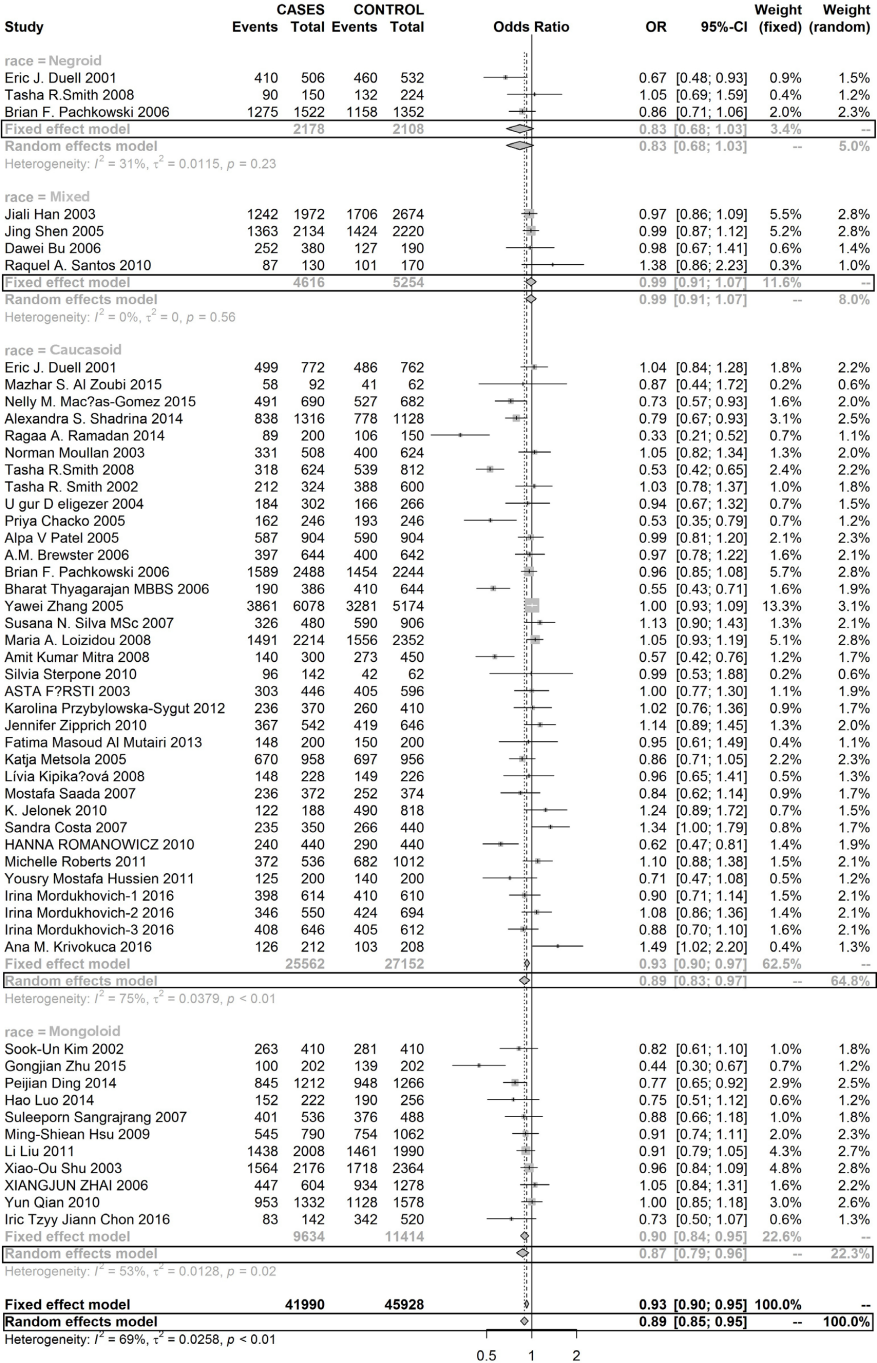
Forest plot of RS25487 polymorphism in XRCC1 and risk to breast cancer (G allele vs. A allele) (the model adopted was marked by black frame)

Three SNPs on the XRCC1 gene were analysed in this meta-analysis. As Supplementary Table 1 show, this meta-analysis didn’t find any evidence to suggest that mutations in rs25489 and rs1799782 were associated with the susceptibility to develop BC in any race population. But, rs25487 with A allele significantly reduced the risk of developing BC in Mongoloid and Caucasoid populations, but not in the Negroid population (Figures 2 and 3).

**Figure 4:**
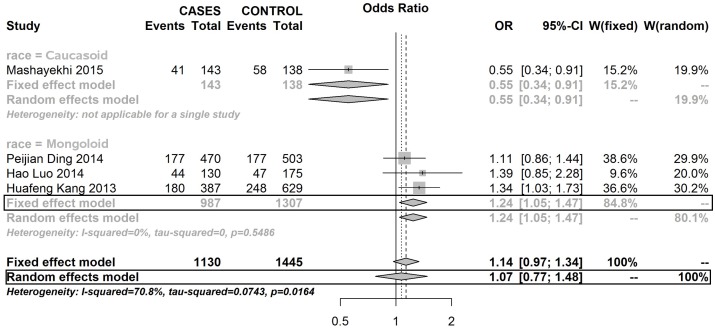
Forest plot of rs1760944 polymorphism in APEX1 and risk to breast cancer (TT vs. CT) (the model adopted was marked by black frame)

**Figure 5:**
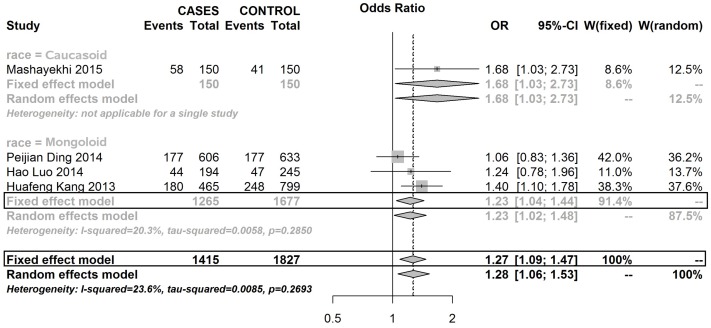
Forest plot of rs1760944 polymorphism in APEX1 and risk to breast cancer (TT vs. CT+CC) (the model adopted was marked by black frame)

Two SNPs on the ADPRT1 gene were analysed in this meta-analysis. As Table 2 shows, there is no evidence which indicates that mutations in SNPs of rs1805414 and rs1136410 were significantly associated with BC. The relationship between two SNPs (rs1130409 and rs1760944) on the APEX1 gene and risk of developing BC was analysed in this meta-analysis (Figures 4 and 5).

**Table 2:** Summary about meta-analysis results of SNPs in ADPRT gene and risk of breast cancer

SNP	Genetic Models	Race	*n*	OR (95% CI)			Homogeneity		*P* for Publication Bias test
				OR	CI	*P* value	Q	I^2^ (%)	
Rs1805414 TT/ CT/ CC	TT + CT vs. CC (Dominant)	Caucasoid	3	0.411	0.134–1.256	0.119	5.000	61.541	0.628
	TT vs. CT + CC (Recessive)	Caucasoid	3	0.576	0.195–1.702	0.318	17.000	88.241	0.758
	T vs. C (Allele)	Caucasoid	3	0.582	0.255–1.33	0.200	16.000	87.383	0.332
	TT vs. CT (Co-Dominant)	Caucasoid	3	0.650	0.207–2.038	0.460	17.000	87.892	0.566
	CT vs. CC (Co-Dominant)	Caucasoid	3	0.519	0.169–1.595	0.252	5.000	57.083	0.659
Rs1136410 CC / CT/ TT	CT+CC vs. TT (Dominant)	Overall	7	1.004	0.787–1.282	0.971	5.000	0.000	0.188
		Caucasoid	6	1.075	0.776–1.487	0.665	4.970	0.000	0.314
	CC vs. CT+ TT	Overall	7	0.996	0.781–1.272	0.971	5.000	0.000	0.683
	(Recessive)	Caucasoid	6	0.931	0.672–1.288	0.665	4.970	0.000	0.547
	C vs. T	Overall	8	0.975	0.822–1.157	0.772	14.000	48.680	0.147
	(Allele)	Caucasoid	6	0.999	0.802–1.247	0.628	11.014	54.605	0.721
	CC vs. CT	Overall	8	0.981	0.811–1.186	0.841	11.000	35.808	0.590
	Co-Dominant	Caucasoid	6	1.021	0.813–1.283	0.857	9.283	40.354	0.918
	CT vs. TT	Overall	7	1.031	0.798–1.331	0.817	4.000	0.000	0.182
	Co-Dominant	Caucasoid	6	1.093	0.778–1.534	0.609	4.087	0.000	0.536

The mutations in rs1130409 did not relate to the risk of developing BC, but T mutated to G in rs1760944 could increase the risk of developing BC in the Mongoloid race. There are insufficient studies which are focused on the relationship of rs1760944 and BC susceptibility outside the Mongoloid population, which restricted the meta-analysis on Mongoloid populations, compared to that done for the Caucasoid and Negroid populations (Table 3).

**Table 3:** Summary about meta-analysis results of SNPs in APEX1 gene and risk of breast cancer

SNP	Genetic Models	Race	*n*	OR (95% CI)			Homogeneity		*P* for Publication Bias
				OR	CI	*P* value	*Q*	I^2^ (%)	
Rs1130409 TT/ TG/ GG	TT + CT vs. CC (Dominant)	Overall	13	1.012	0.773–1.325	0.932	74.000	83.730	0.412
		Caucasoid	7	0.942	0.604–1.469	0.794	60.567	90.094	0.778
		Mongoloid	5	1.076	0.779–1.486	0.658	13.195	69.684	0.747
	TT vs. CT+CC	Overall	13	0.918	0.783–1.075	0.288	37.000	67.181	0.703
	(Recessive)	Caucasoid	7	0.840	0.623–1.132	0.251	30.948	80.613	0.144
		Mongoloid	5	0.966	0.837–1.115	0.637	5.348	25.199	0.342
	T vs. C	Overall	13	0.967	0.831–1.124	0.658	75.000	83.977	0.363
	(Allele)	Caucasoid	7	0.908	0.688–1.199	0.4-95	69.148	91.323	0.786
		Mongoloid	5	1.005	0.903–1.118	0.928	5.635	29.015	0.073
	TT vs. CT	Overall	13	0.878	0.753–1.026	0.101	30.000	60.592	0.133
	Co-Dominant	Caucasoid	7	0.799	0.61–1.047	0.104	21.625	72.254	0.739
		Mongoloid	5	0.937	0.775–1.133	0.503	8.105	50.650	0.295
	CT vs. CC	Overall	13	1.074	0.812–1.421	0.615	69.000	82.658	0.944
	Co-Dominant	Caucasoid	7	1.042	0.665–1.633	0.858	53.503	88.786	0.978
		Mongoloid	5	1.111	0.764–1.614	0.582	15.600	74.359	0.972
Rs1760944 TT/ CT /CC	TT+CT vs. CC	Overall	4	1.000	0.840–1.192	0.996	7.000	55.865	0.899
	(Dominant)	Mongoloid	3	1.021	0.854–1.221	0.816	5.387	62.873	0.609
	TT vs. CT+CC	Overall	4	1.265	1.086–1.474	0.003	4.000	23.638	0.763
	(Recessive)	Mongoloid	3	1.227	1.044–1.441	0.013	2.511	20.344	0.943
	T vs. C	Overall	4	1.106	1.001–1.222	0.049	6.000	50.892	0.638
	(Allele)	Mongoloid	3	1.098	0.989–1.219	0.081	5.909	66.153	0.609
	TT vs. CT	Overall	4	1.067	0.772–1.478	0.122	3.000	4.848	0.359
	Co-Dominant	Mongoloid	3	1.241	1.045–1.473	0.014	1.201	0.000	0.727
	CT vs. CC	Overall	4	0.913	0.757–1.101	0.337	6.000	46.736	0.882
	Co-Dominant	Mongoloid	3	0.938	0.775–1.135	0.511	3.641	45.072	0.279

**Figure 6:**
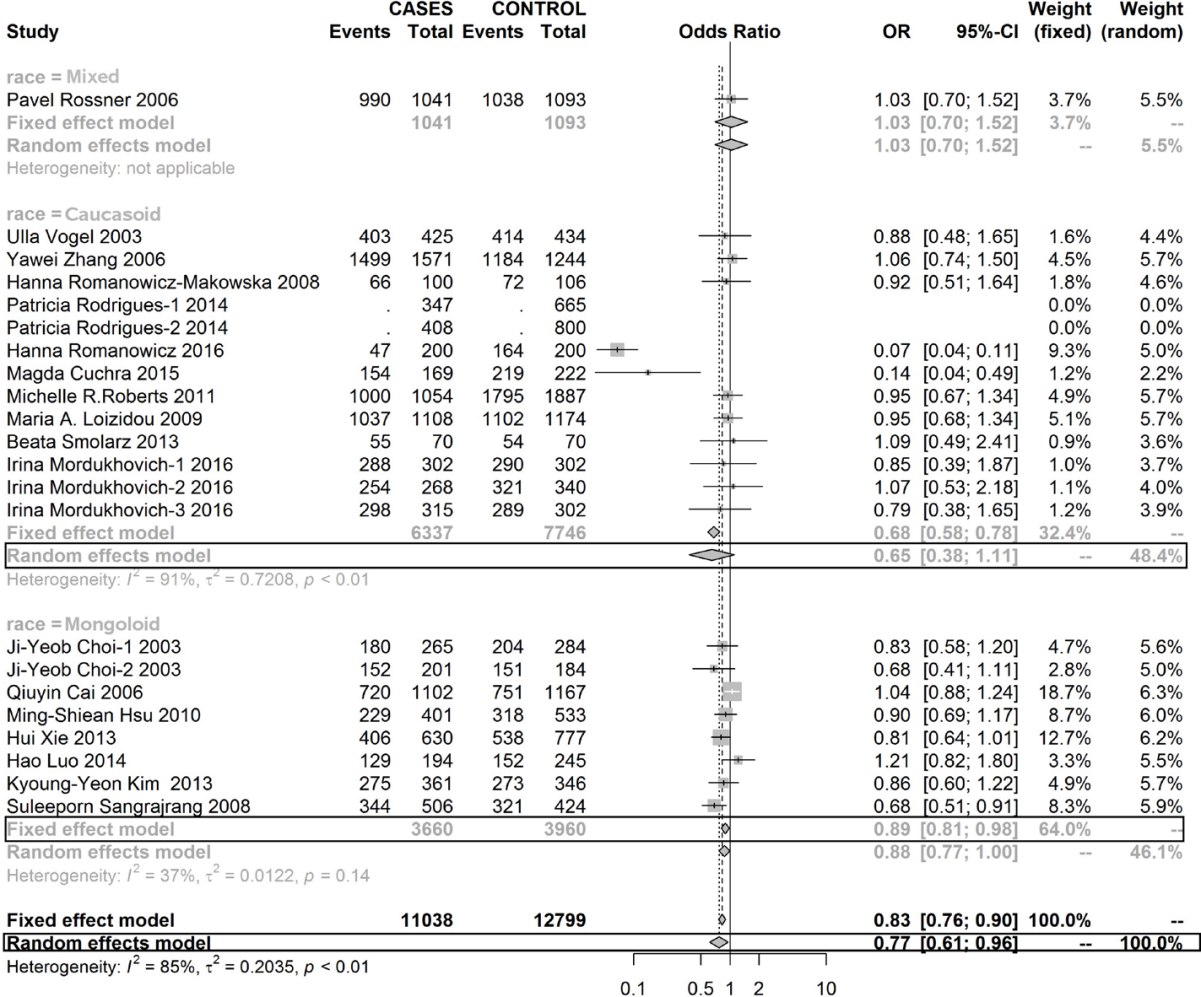
Forest plot of rs1052133 polymorphism in OGG1 and risk to breast cancer (CC+CG vs. GG) (the model adopted was marked by black frame)

**Figure 7:**
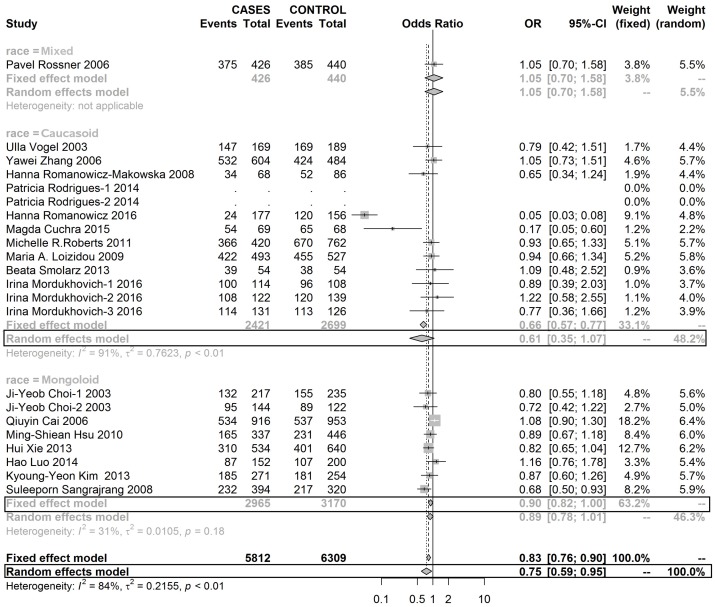
Forest plot of rs1052133 polymorphism in OGG1 and thus risk to breast cancer (CG vs. GG) (the model adopted was marked by black frame)

**Figure 8:**
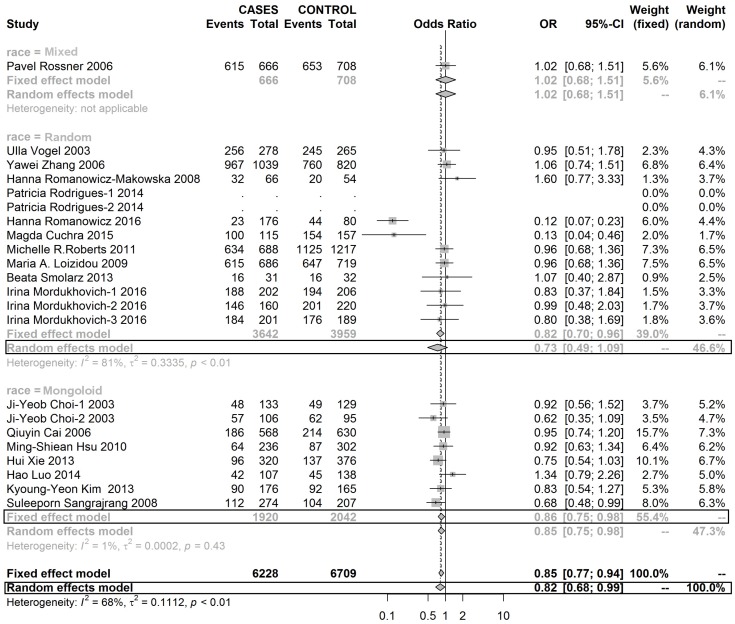
Forest plot of rs1052133 polymorphism in OGG1 and thus risk to breast cancer (CC vs. GG) (the model adopted was marked by black frame)

Table 4 shows a CC genotype of rs1052133 played a protective role in the development of BC in Mongoloid and Caucasoid race populations (Figures 6, 7 and 8).

**Table 4:** Summary about meta-analysis results of SNPs in OGG1 gene and risk of breast cancer

SNP	Genetic Models	Race	*n*	OR(95% CI)			Homogeneity		*P* for Publication Bias
				OR	CI	*P* value	*Q*	I^2^ (%)	
rs1052133 CC/ CG/ GG	CC+CG vs. GG (Dominant)	Overall	20	0.767	0.611–0.962	0.021	128	85.21	0.478
		Caucasoid	11	0.646	0.376–1.107	0.112	111.303	91.016	0.735
		Mongoloid	8	0.877	0.770–0.998	0.047	11.056	36.687	0.425
	CC vs .CG+GG	Overall	22	1.215	0.819–1.802	0.334	133	84.168	0.665
	(Recessive)	Caucasoid	13	1.224	0.962–1.556	0.100	112.479	89.331	0.139
		Mongoloid	8	0.955	0.749–1.217	0.709	11.056	36.687	0.391
	C vs. G	Overall	20	1.002	0.897–1.119	0.968	115	83.47	0.841
	(Allele)	Caucasoid	11	0.994	0.818–1.208	0.954	87.007	88.507	0.211
		Mongoloid	8	1.006	0.880–1.149	0.932	26.557	73.642	0.480
	CC vs.CG	Overall	20	0.818	0.677–0.987	0.036	59	67.958	0.488
	Co-Dominant	Caucasoid	11	0.734	0.492–1.093	0.128	51.521	80.591	0.577
		Mongoloid	8	0.855	0.749–0.976	0.020	7.037	0.521	0.583
	CG vs. GG	Overall	20	0.752	0.595–0.951	0.017	122	84.455	0.289
	Co-Dominant	Caucasoid	11	0.613	0.352–1.070	0.085	105.605	90.531	0.131
		Mongoloid	8	0.886	0.777–1.010	0.052	10.097	30.673	0.735

Sensitivity analysis demonstrated all the results were robust, and no significant publication bias was found.

## DISCUSSION

This study quantitatively summarized the association between 8 SNPs on 4 BER genes and the risk of developing BC, by pooling the data from 69 papers with 33760 BC cases and 33252 control individuals. To the best of our knowledge, this paper is novel in its discussion in terms of the difference to the susceptibility to BC in different races. Overall, the results of this paper found that the mutations of rs25487 on the XRCC1 gene, rs1760944 on APEX1 and rs1052133 on the OGG1 gene were significantly related with susceptibility to BC.

Rs25487 on the XRCC1 gene (also known as Gln399Arg, and A allele encodes the Gln amino acid) was the polymorphism most studied in the risk of cancers. Rs25487 participates in coding of BRCT I domains of XRCC 1, which is one of the interaction domains of BRCA 1 protein [79]. BRCA 1 has been proved to be a predict gene for hereditary BC, it can suppress developing of breast cancer in humans [80, 81] This meta-analysis found populations with A allele could significantly reduce the risk of developing BC. Similar findings are presented in other cancers studies. For instance, in two studies carried on Americans and Koreans, the rs25487(A/A) genotype significantly reduces the risk of both basal cell and squamous cell cancers [82, 83]. A meta-analysis found the G allele is one of the risk factors in developing of Glioma in Asians [84]. Another meta-analysis carried out on a Chinese population found XRCC1 Arg399Gln polymorphism is not associated with BC (at the 5% level) but indicated a borderline association [85].

In human cells, APE1 gene, located on chromosome 14, encodes the primar AP endonuclease. AP sites is frequently happened in DNA molecules, which can help cells to recognize and repair DNA damage [86]. Rs1130409 and rs1760944 on the APE1 gene has been widely researched with respect to its role in cancer susceptibility [87–89]. By pooling 4 studies (3 on Chinese populations and 1 on an Iran population), this meta-analysis found the rs1760944 (also known as – 656T > G) variants was associated with an incaresed risk of developing BC in Mongoloid populations, or more specifically, in the Chinese population. Rs1760944 polymorphism is located on Polymorphisms in a promoter region and is located −141bp upstream from the transcription initiation site. Variants in the promoter region, or 3’UTR, of a gene may influence its function and lead to abnormal protein expression. Function studies have proved that Rs1760944 mutation can influence its activity of communicating to other BER proteins [69].

The key role of OGG1 protein is to cleave 8-hydroxyguanine. [90] Rs1052133, also known as Ser326Cys, is a SNP on the OGG1 gene, and the minor (G) allele, encoding the cysteine. This paper found that in contrast to the GG genotype, people who carried the CC or CG genotype had a lower risk of developing BC in both the Mongoloid and Caucasoid race populations. This study indicated that rs1052133 may follow a recessive inheritance pattern in BC susceptibility, because the OR of CC vs. GG genotypes was similar to CG vs. GG genotypes (results shown in Table 4). Lee et al. (2015) found that ability of oxidative DNA damage repair was significant lower in GG genotype individuals than non-GG genotype individuals [91].

## CONCLUSIONS

This study suggests that rs1052133, rs25487 and rs1760944 polymorphisms may influence individual susceptibility to risk of developing BC, and provides evidence which supports the idea that mutations of the DNA repair genes are associated to BC risk. The understanding of BC etiology and roles in the potential biological pathways linking DNA repair, ethnic background, environment and BC need to be studied further.

## SUPPLEMENTARY MATERIALS TABLES




