# Supplementary material for: Polymorphisms in BER genes and risk of breast cancer: evidences from 69 studies with 33760 cases and 33252 controls
Source: Oncotarget. 2018 Jan 2;9(22):16220–33. doi: 10.18632/oncotarget.23804 (PMC5882330; doi:10.18632/oncotarget.23804)
Supplement: Supplementary file 1 [file oncotarget-09-16220-s001.pdf]

# Polymorphisms in BER genes and risk of breast cancer: evidences from 69 studies with 33760 cases and 33252 controls

## SUPPLEMENTARY MATERIALS

**Supplementary Table 1: Summary about meta-analysis results of SNPs in XRCC1 gene and risk of breast cancer.** See_Supplementary Table_1
