# Supplementary material for: Polymorphisms in BER genes and risk of breast cancer: evidences from 69 studies with 33760 cases and 33252 controls
Source: Oncotarget. 2018 Jan 2;9(22):16220–33. doi: 10.18632/oncotarget.23804 (PMC5882330; doi:10.18632/oncotarget.23804)
Supplement: Supplementary file 2 [file oncotarget-09-16220-s002.docx]

**Supplementary Table 1: Summary about meta-analysisresults of SNPs in XRCC1 gene and risk of breast cancer**

| SNP | Genetic  Models | Race | n | OR(95% CI) | | | Homogeneity | | *P* for Publication  Bias |
| --- | --- | --- | --- | --- | --- | --- | --- | --- | --- |
|  |  |  |  | OR | CI | P value | Q | I²(%) |  |
| Rs1799782  CC/CT/ TT | CT+CC vs. TT  (Dominant) | Overall | 33 | 0.946 | 0.812-1.102 | 0.477 | 16.000 | 0.000 | 0.653 |
|  |  | Negroid | 3 | 1.569 | 0.382-6.444 | 0.532 | 1.158 | 0.000 | 0.694 |
|  |  | Mixeded | 1 | 0.728 | 0.147-3.616 | 0.698 | 0.000 | - | 0.931 |
|  |  | Caucasoid | 21 | 0.770 | 0.564-1.051 | 0.100 | 7.116 | 0.000 | 0.528 |
|  |  | Mongoloid | 8 | 1.007 | 0.843-1.202 | 0.941 | 5.254 | 0.000 | 0.432 |
|  | CC vs. CT+TT | Overall | 38 | 0.964 | 0.873-1.065 | 0.470 | 82.00 | 54.924 | 0.828 |
|  | (Recessive) | Negroid | 3 | 1.070 | 0.825-1.387 | 0.611 | 1.263 | 0.000 | 0.637 |
|  |  | Mixed | 2 | 1.155 | 0.965-1.382 | 0.117 | 0.601 | 0.000 | 0.611 |
|  |  | Caucasoid | 25 | 0.896 | 0.759-1.058 | 0.196 | 68.335 | 64.879 | 0.158 |
|  |  | Mongoloid | 8 | 1.040 | 0.945-1.144 | 0.425 | 6.227 | 0.000 | 0.255 |
|  | C vs. T | Overall | 33 | 0.954 | 0.870-1.047 | 0.321 | 70.000 | 54.422 | 0.701 |
|  | (Allele) | Negroid | 3 | 1.080 | 0.844-1.383 | 0.539 | 1.350 | 0.000 | 0.062 |
|  |  | Mixed | 1 | 1.209 | 0.948-1.542 | 0.126 | 0.000 | - | 0.716 |
|  |  | Caucasoid | 21 | 0.875 | 0.743-1.030 | 0.108 | 55.560 | 64.003 | 0.238 |
|  |  | Mongoloid | 8 | 1.026 | 0.951-1.107 | 0.500 | 6.430 | 0.000 | 0.742 |
|  | CC vs. CT | Overall | 33 | 0.964 | 0.858-1.084 | 0.543 | 79.000 | 59.509 | 0.895 |
|  | Co-Dominant | Negroid | 3 | 1.054 | 0.809-1.373 | 0.697 | 1.248 | 0.000 | 0.312 |
|  |  | Mixed | 1 | 1.254 | 0.97-1.6210 | 0.085 | 0.000 | - | 0.447 |
|  |  | Caucasoid | 21 | 0.892 | 0.727-1.095 | 0.276 | 67.069 | 70.180 | 0.690 |
|  |  | Mongoloid | 8 | 1.039 | 0.94-1.149 | 0.450 | 5.510 | 0.000 | 0.809 |
|  | CT vs. TT | Overall | 33 | 0.936 | 0.798-1.099 | 0.421 | 17.000 | 0.000 | 0.100 |
|  | Co-Dominant | Negroid | 3 | 1.462 | 0.348-6.151 | 0.604 | 1.245 | 0.000 | 0.880 |
|  |  | Mixed | 1 | 0.597 | 0.118-3.01 | 0.532 | 0.000 | - | 0.983 |
|  |  | Caucasoid | 21 | 0.791 | 0.571-1.095 | 0.158 | 8.882 | 0.000 | 0.231 |
|  |  | Mongoloid | 8 | 0.988 | 0.820-1.192 | 0.902 | 4.454 | 0.000 | 0.810 |
| RS25487  GG/ GA/ AA | GA+GG vs. AA | Overall | 53 | 0.788 | 0.691-0.898 | <0.001 | 204 | 74.479 | 0.868 |
|  | (Dominant) | Negroid | 3 | 0.776 | 0.513-1.231 | 0.355 | 2.706 | 26.101 | 0.526 |
|  |  | Mixed | 4 | 1.031 | 0.859-1.215 | 0.810 | 2.684 | 0.000 | 0.387 |
|  |  | Caucasoid | 35 | 0.799 | 0.669-0.954 | 0.013 | 166.758 | 79.611 | 0.161 |
|  |  | Mongoloid | 11 | 0.688 | 0.547-0.865 | 0.001 | 23.012 | 56.545 | 0.078 |
|  | GG vs. GA+AA | Overall | 53 | 0.919 | 0.863-0.979 | 0.009 | 112 | 53.759 | 0.114 |
|  | (Recessive) | Negroid | 3 | 0.812 | 0.649-1.016 | 0.069 | 2.487 | 19.577 | 0.128 |
|  |  | Mixed | 4 | 0.964 | 0.859-1.082 | 0.533 | 1.61 | 0.000 | 0.712 |
|  |  | Caucasoid | 35 | 0.926 | 0.851-1.008 | 0.077 | 78.869 | 56.890 | 0.551 |
|  |  | Mongoloid | 11 | 0.89 | 0.772-1.025 | 0.107 | 27.252 | 63.305 | 0.054 |
|  | G vs. A | Overall | 53 | 0.894 | 0.845-0.947 | <0.001 | 167 | 68.875 | 0.313 |
|  | (Allele) | Negroid | 3 | 0.835 | 0.676-1.030 | 0.092 | 2.916 | 31.412 | 0.776 |
|  |  | Mixed | 4 | 0.987 | 0.908-1.072 | 0.753 | 2.051 | 0.000 | 0.579 |
|  |  | Caucasoid | 35 | 0.895 | 0.826-0.969 | 0.006 | 135.812 | 74.965 | 0.860 |
|  |  | Mongoloid | 11 | 0.870 | 0.790-0.958 | 0.005 | 21.396 | 53.262 | 0.218 |
|  | GG vs. GA | Overall | 53 | 0.967 | 0.898-1.042 | 0.382 | 140 | 62.742 | 0.074 |
|  | Co-Dominant | Negroid | 3 | 0.814 | 0.643-1.031 | 0.088 | 2.405 | 16.834 | 0.611 |
|  |  | Mixed | 4 | 0.956 | 0.848-1.079 | 0.470 | 1.186 | 0.000 | 0.371 |
|  |  | Caucasoid | 35 | 0.988 | 0.891-1.096 | 0.818 | 103.99 | 67.305 | 0.310 |
|  |  | Mongoloid | 11 | 0.945 | 0.809-1.105 | 0.481 | 29.512 | 66.116 | 0.715 |
|  | GA vs. AA | Overall | 53 | 0.795 | 0.687-0.919 | 0.002 | 222 | 76.545 | 0.647 |
|  | Co-Dominant | Negroid | 3 | 0.843 | 0.475-1.496 | 0.560 | 2.340 | 14.530 | 0.258 |
|  |  | Mixed | 4 | 1.047 | 0.873-1.256 | 0.621 | 2.331 | 0.000 | 0.180 |
|  |  | Caucasoid | 35 | 0.796 | 0.652-0.971 | 0.024 | 183.751 | 81.497 | 0.099 |
|  |  | Mongoloid | 11 | 0.701 | 0.545-0.900 | 0.005 | 24.919 | 59.871 | 0.170 |
| RS25489  GG/ GA/ AA | GA+GG vs. AA | Overall | 10 | 0.874 | 0.621-1.231 | 0.442 | 9.000 | 0.472 | 0.925 |
|  | (Dominant) | Negroid | 1 | 1.124 | 0.07-17.997 | 0.934 | 0.000 | - | 0.687 |
|  |  | Caucasoid | 8 | 0.965 | 0.658-1.415 | 0.856 | 7.054 | 0.768 | 0.442 |
|  |  | Mongoloid | 1 | 0.501 | 0.213-1.175 | 0.112 | 0.000 | - | 0.515 |
|  | GG vs. GA+AA | Overall | 10 | 1.144 | 0.812-1.611 | 0.442 | 9.000 | 0.472 | 0.680 |
|  | (Recessive) | Negroid | 1 | 0.890 | 0.056-14.257 | 0.934 | 0.000 | - | 0.294 |
|  |  | Caucasoid | 8 | 1.036 | 0.706-1.521 | 0.856 | 7.054 | 0.768 | 0.311 |
|  |  | Mongoloid | 1 | 1.998 | 0.851-4.692 | 0.112 | 0.000 | - | 0.729 |
|  | A vs. G | Overall | 11 | 0.959 | 0.871-1.056 | 0.397 | 11.000 | 11.103 | 0.954 |
|  | (Allele) | Negroid | 2 | 0.818 | 0.552-1.212 | 0.316 | 0.174 | 0.000 | 0.077 |
|  |  | Caucasoid | 8 | 0.973 | 0.855-1.106 | 0.672 | 10.290 | 31.974 | 0.940 |
|  |  | Mongoloid | 1 | 0.941 | 0.767-1.155 | 0.560 | 0.000 | - | 0.337 |
|  | GG vs. GA | Overall | 11 | 0.971 | 0.857-1.12 | 0.644 | 14.000 | 29.450 | 0.402 |
|  | Co-Dominant | Negroid | 2 | 0.802 | 0.534-1.205 | 0.289 | 0.197 | 0.000 | 0.272 |
|  |  | Caucasoid | 8 | 0.980 | 0.832-1.154 | 0.810 | 12.922 | 45.828 | 0.422 |
|  |  | Mongoloid | 1 | 1.022 | 0.813-1.284 | 0.853 | 0.000 | - | 0.129 |
|  | GA vs. AA | Overall | 10 | 0.799 | 0.518-1.233 | 0.311 | 10.000 | 10.023 | 0.182 |
|  | Co-Dominant | Negroid | 1 | 1.421 | 0.086-23.432 | 0.806 | 0.000 | - | 0.872 |
|  |  | Caucasoid | 8 | 0.860 | 0.503-1.471 | 0.583 | 8.043 | 12.971 | 0.490 |
|  |  | Mongoloid | 1 | 0.492 | 0.205-1.178 | 0.111 | 0.000 | - | 0.868 |
